# Supplementary material for: Plastomes of Bletilla (Orchidaceae) and Phylogenetic Implications
Source: Int J Mol Sci. 2022 Sep 5;23(17):10151. doi: 10.3390/ijms231710151 (PMC9456473; doi:10.3390/ijms231710151)
Supplement: Supplementary file 1 [file ijms-23-10151-s001.zip › FigS1.pdf]

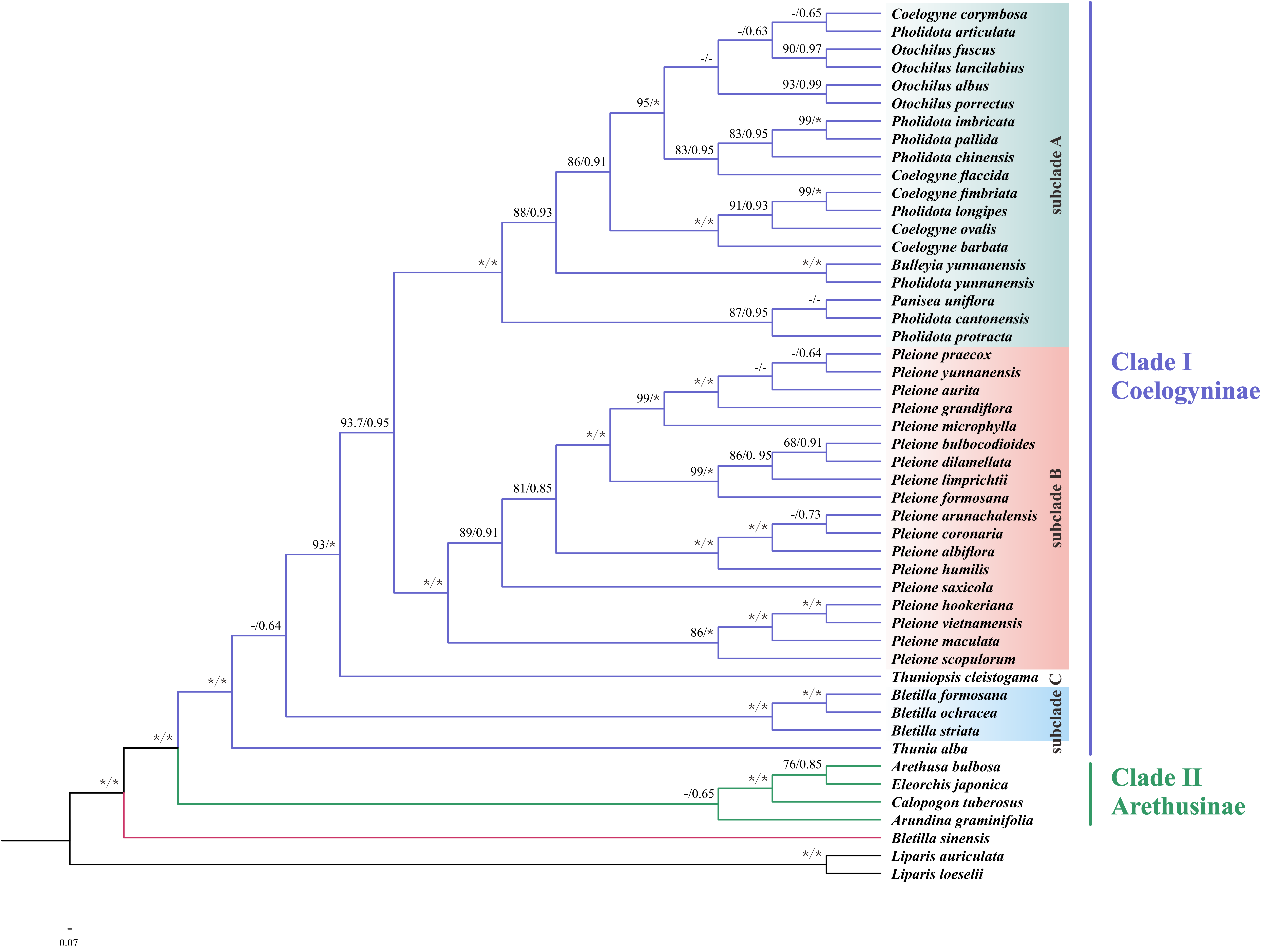

Figure S1. The phylogenetic tree of 49 Arethuseae species based on six cpDNA loci using the maximum likelihood (ML) and the Bayesian inference (BI) method. The bootstrap (BS) and Bayesian poste-rior probability (PP) values of each node were labeled (* denoted 100% bootstrap or 1.00 PP, with the omission of those <50% bootstrap or <0.5 PP).
